# Effects of Gelatin Hydrolysate from Bigeye Snapper (*Priacanthus tayenus*) Skin in Mitigating Oxidative Stress in Chronic Cerebral Hypoperfusion Rats

**DOI:** 10.3390/ijms27062856

**Published:** 2026-03-21

**Authors:** Jirakhamon Sengking, Phakkawat Thangwong, Pranglada Jearjaroen, Nuttapong Yawoot, Sutee Wangtueai, Jiraporn Tocharus, Chainarong Tocharus

**Affiliations:** 1 Department of Anatomy, Faculty of Medicine, Chiang Mai University, Chiang Mai 50200, Thailand; nnsengking@gmail.com; 2 Department of Medical Science, School of Medicine, Walailak University, Nakhon Si Thammarat 80160, Thailand; natphakkawat@gmail.com; 3 Research Center in Tropical Pathology, Walailak University, Nakhon Si Thammarat 80160, Thailand; 4 Integrated Neuro-Musculoskeletal, Chronic Disease, and Aging Research Engagement Center (ICARE Center), Department of Physical Therapy, Faculty of Associated Medical Sciences, Chiang Mai University, Chiang Mai 50200, Thailand; pranglada@gmail.com; 5 Department of Physiology, Faculty of Medical Science, Naresuan University, Phitsanulok 65000, Thailand; nuttapong.yw@gmail.com; 6 School of Agro-Industry, Faculty of Agro-Industry, Chiang Mai University, Chiang Mai 50100, Thailand; sutee.w@cmu.ac.th; 7 Cluster of Innovation for Sustainable Seafood Industry and Value Chain Management, Chiang Mai University, Chiang Mai 50200, Thailand; 8 Department of Physiology, Faculty of Medicine, Chiang Mai University, Chiang Mai 50200, Thailand; jiraporn.tocharus@cmu.ac.th

**Keywords:** gelatin hydrolysate, chronic cerebral hypoperfusion, cognitive impairment, oxidative stress, neuronal apoptosis

## Abstract

Gelatin hydrolysate (GH), a bioactive compound derived from collagen, has demonstrated potential therapeutic benefits in various medical conditions. However, its effects on chronic cerebral hypoperfusion-induced vascular dementia remain underexplored. This study aimed to investigate the anti-oxidative stress effects of GH in alleviating brain damage and cognitive impairment in CCH-induced rats. Male Wistar rats underwent bilateral common carotid artery occlusion to induce CCH and were randomly divided into five groups: (1) sham, (2) 2-vessel occlusion (2VO), (3) 2VO + 250 mg/kg GH, (4) 2VO + 500 mg/kg GH, and (5) 2VO + piracetam. Treatments were administered for 35 days of post-operation. GH treatment significantly mitigated oxidative stress, as evidenced by reduced levels of reactive oxygen species (ROS), nitric oxide (NO), and the expression of 4-hydroxynonenal (4-HNE) and NADPH oxidase 4 (NOX4). Furthermore, GH exhibited antioxidant activity by upregulating superoxide dismutase (SOD) levels via nuclear factor E2-related factor 2 (Nrf-2) activation. This, in turn, reduced neuronal apoptosis by decreasing Bax and cleaved-caspase 3 levels and increasing Bcl-2 expression. Additionally, GH treatment ameliorated Tau protein hyperphosphorylation and improved synaptic function. Overall, GH exerted neuroprotective effects against oxidative stress-related neuronal damage and enhanced neuroplasticity, learning, and memory in rats with CCH-induced cognitive impairment.

## 1. Introduction

Vascular dementia (VaD) is the second-most prevalent form of dementia after Alzheimer’s disease (AD) and is commonly caused by impaired blood supply to the brain [1]. Clinically, approximately 15–20% of VaD cases arise from cerebrovascular pathologies that lead to chronic cerebral hypoperfusion (CCH) [1,2,3]. This condition results in reduced cerebral blood flow, which contributes to progressive neuronal damage and cognitive decline [2]. Previous studies have reported that vascular pathologies are significant risk factors for AD by promoting beta-amyloid (Aβ) accumulation and tau protein (pTau) hyperphosphorylation, both of which contribute to cognitive impairment and have been observed in preclinical models [3,4]. In addition, oxidative stress is one of the key factors contributing to the pathogenesis of CCH-induced VaD [2]. During CCH, oxidative stress plays a critical role by increasing reactive oxygen species (ROS) and reducing the brain’s antioxidant defense mechanisms [5]. Previous studies have shown that elevated oxidative stress triggers lipid peroxidation, leading to the formation of 4-hydroxynonenal (4-HNE), DNA damage, and ultimately, neuronal apoptosis [6,7]. Apoptosis is a hallmark of neurodegenerative diseases [8]. The resulting reduction in neuronal cell numbers, combined with oxidative damage, primarily disrupts synaptic plasticity [8,9,10]. Synaptic plasticity refers to the capacity of synapses to modify their strength and efficiency in response to neuronal activity, which is essential for learning and memory. However, previous studies have reported that CCH disrupts synaptic functions, contributing to cognitive impairment [11,12]. Therefore, therapeutic approaches that target the interplay between CCH-induced oxidative stress and its associated pathological mechanisms may be effective in attenuating cognitive decline in CCH-induced VaD.

Fish gelatin hydrolysate (GH), also known as hydrolyzed fish gelatin, is a highly versatile protein and peptide derived from fish byproducts, especially collagenous materials such as fish skin, bone, and scales [13,14,15].

Fish GH is the sequencing of a mixture of amino acids, and these smaller peptides are more easily digested, soluble in cold water, and have a wide range of health-beneficial properties [13,16]. Recent evidence also suggests that a collagen-rich environment can promote the effective function of brain-derived neurotrophic factor (BDNF) by providing the necessary structural support, thereby enhancing synaptic plasticity and neuroprotection [17,18,19]. In addition, a study has shown that collagen-derived derivatives have significant antioxidant properties [20].

Since the role of GH in terms of its effect in the brain has not yet been elucidated and the benefits of fish GH in CCH-induced VaD have not been reported. Therefore, in the present study, we investigated the neuroprotective role of fish GH in CCH-induced VaD and explored the underlying mechanism of action of the neuroprotective properties of fish GH.

## 2. Results

### 2.1. GH Improved Cognitive Function in CCH Rats

**Figure 1:**
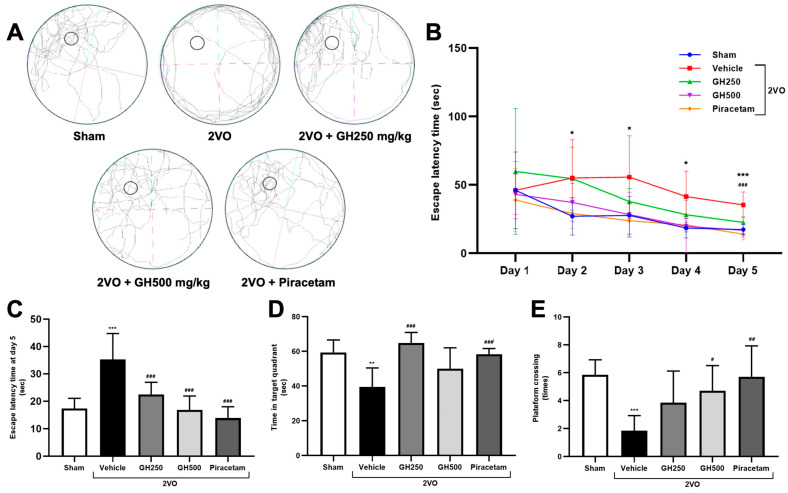
Gelatin hydrolysate improves cognitive impairment in 2VO rats, as assessed by the MWM test. (**A**) The tracking for the probe trial day by the SMART program. (**B**) The escape latency time from days 1–5. (**C**) The escape latency time on day 5. (**D**) Time in the target quadrant on day 6. (**E**) The number of platform crossings on day 6. 2VO rats exhibited significantly prolonged escape latencies, decreased time spent in the target quadrant, and reduced platform crossing. Treatment with GH at 250, 500 mg/kg, and piracetam significantly improved learning performance, as demonstrated by reduced escape latency, increased time spent in the target quadrant, and increased platform crossing. Data are presented as mean ± SD. (*n* = 6 per group). Statistical analysis was performed using two-way ANOVA followed by a Bonferroni test. * *p* < 0.05, ** *p* < 0.01, and *** *p* < 0.001 indicate a significant difference from the sham-operated group, and ^#^ *p* < 0.05, ^##^ *p* < 0.01, and ^###^ *p* < 0.001 denote a significant difference relative to the 2VO + vehicle group.

Cognitive impairment under the CCH condition was demonstrated using the MWM test. Rats in the 2VO group showed learning and memory impairment compared to the sham group as indicated by significantly longer escape latency, decreased platform crossing, and time in the target quadrant (Figure 1A–E). GH at the doses of 250 mg/kg, 500 mg/kg, or piracetam-treated rats significantly time to reach the platform (*p* < 0.001) compared to the sham group. A significant increase in time spent in the target quadrant was shown in GH at the dose of 250 mg/kg and piracetam treatment (*p* < 0.001), compared to the 2VO group. While GH at the dose of 500 mg/kg tended to increase the time spent in the target quadrant. For platform crossing, 500 mg/kg GH and piracetam showed significantly increased times of crossing (*p* < 0.05 and *p* < 0.01, respectively) in comparison with the 2VO rats (Figure 1E). These results demonstrated an improvement in cognitive function in GH-treated rats.

### 2.2. GH Attenuated Hypoxic Condition and Oxidative Stress in CCH Rats

**Figure 2:**
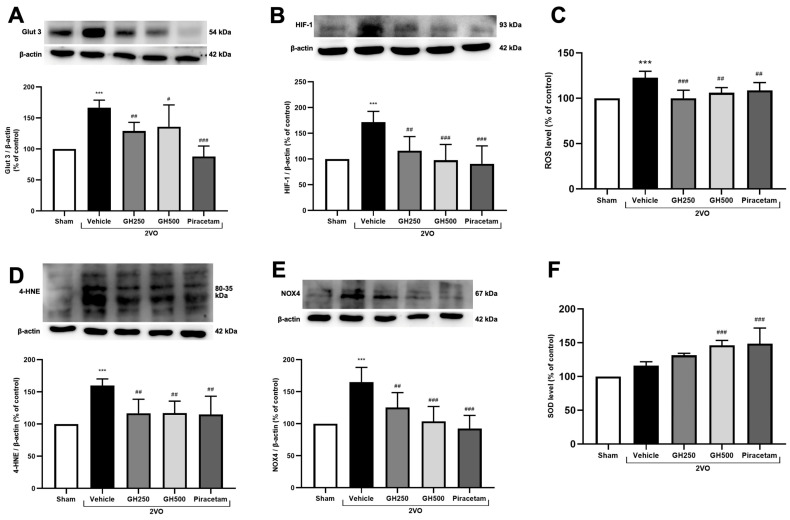
Effects of gelatin hydrolysate on hypoxic conditions and oxidative stress in CCH rats. Western blotting was conducted to determine the protein expression of Glut 3, HIF-1, 4HNE, and NOX4 in the rat whole brain following 2VO. The comparison includes the sham, 2VO + vehicle, 2VO + GH (250 mg/kg BW), 2VO + GH (500 mg/kg BW), and 2VO + piracetam group (**A**) Representative Western blot analysis band and quantification of Glut 3 expression, normalized to β-actin. (**B**) Representative Western blot analysis band and quantification of HIF-1expression, normalized to β-actin. (**C**) ROS level. (**D**) Representative Western blot analysis band and quantification of 4-HNE expression, normalized to β-actin. (**E**) Representative Western blot analysis band and quantification of NOX4 expression, normalized to β-actin. (**F**) SOD activity. All data are presented as mean ± SD. (*n* = 6 per group). *** *p* < 0.001 indicates a significant difference from the sham-operated group, and ^#^ *p* < 0.05, ^##^ *p* < 0.01, ^###^ *p* < 0.001 denote a significant difference relative to the 2VO + vehicle group.

GLUT3, a neuronal glucose transporter, and HIF-1, an oxygen sensor, are upregulated under CCH conditions. The expression levels of GLUT3 and HIF-1 were evaluated using Western blot analysis. In 2VO rats, the levels of GLUT3 and HIF-1 were significantly increased compared to the sham group, confirming the induction of CCH by the 2VO model. Treatment with GH or piracetam significantly decreased GLUT3 and HIF-1 protein expression compared to 2VO rats (Figure 2A,B).

The levels of ROS and 4-HNE in brain tissues were assessed as indicators of oxidative stress under CCH conditions. Compared to the sham group, 2VO rats exhibited significantly elevated levels of both ROS and 4-HNE, a highly toxic end-product of lipid peroxidation. Treatment with GH (250 or 500 mg/kg) or piracetam markedly reduced ROS and 4-HNE levels compared to the 2VO rats (Figure 2C,D). Additionally, the expression of NOX4, a major enzyme responsible for superoxide production in the brain, was significantly upregulated in the 2VO group relative to the sham group. GH or piracetam treatment significantly decreased NOX4 expression compared to the 2VO rats (Figure 2E).

Next, the activity of SOD, a key antioxidant enzyme, was assessed. A significant increase in SOD activity was observed in rats treated with GH at a dose of 500 mg/kg or with piracetam, compared to the 2VO group. These findings suggest that GH exerts antioxidant effects in 2VO rats (Figure 2F).

### 2.3. Effects of GH on Apoptotic Cell Death Under CCH Conditions

**Figure 3:**
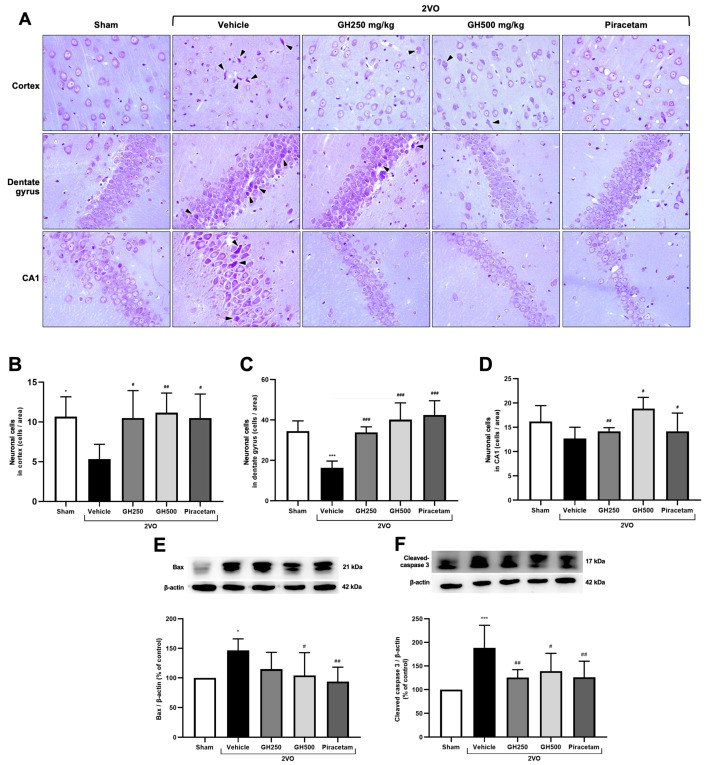
Effects of gelatin hydrolysate against apoptotic cell death under CCH conditions. Photomicrographs of brain sections stained with: (**A**) Nissl staining micrograph of the cortex and hippocampal region, black arrowheads showing shrinkage and pyknotic neurons, 400× total magnification. The image was visualized under a light microscope (scale bar = 50 μm). (**B**) Quantification of the total number of neurons in the cortical area. (**C**) Quantification of the total number of neurons in the dentate gyrus area of the hippocampus. (**D**) Quantification of the total number of neurons in the CA1 area of the hippocampus. (**E**) Representative Western blot analysis band and quantification of the expression of Bax, normalized to β-actin. (**F**) Representative Western blot analysis band and quantification of the expression of cleaved-caspase 3, normalized to β-actin. All data are presented as mean ± SD. (*n* = 6 per group). * *p* < 0.05, and *** *p* < 0.001 indicate a significant difference from the sham-operated group, and ^#^ *p* < 0.05, ^##^ *p* < 0.01, and ^###^ *p* < 0.001 denote a significant difference relative to the 2VO + vehicle group.

To evaluate the protective effects of GH against apoptosis induced by CCH, apoptotic markers and histological analyses were examined. The 2VO rats exhibited neuronal shrinkage with pyknotic nuclei and surrounding vacuolization (indicated by black arrowheads) in both the cortex and hippocampus, compared to the sham group. Notably, these changes were markedly improved in the GH or piracetam-treated groups, where neuronal integrity in the cortex and hippocampus was significantly preserved (Figure 3A). The number of healthy neurons was quantified in the cortex, dentate gyrus, and CA1 regions. A marked reduction in neuronal cells was observed in the CA1 region in 2VO rats. A significant decrease was also detected in the cortex and dentate gyrus compared with the normal control group (Figure 3B–D). Treatment with GH and piracetam increased the number of neuronal cells across all examined regions compared with the 2VO group. We next assessed the expression of Bax and cleaved caspase 3, key markers of apoptotic cell death, via Western blotting. The results showed upregulation of both Bax and cleaved caspase-3 in the 2VO rats compared to the sham group (Figure 3E,F). However, treatment with GH or piracetam significantly reduced these apoptotic markers compared to the 2VO group.

### 2.4. GH Ameliorated AChE Activity, Tau Protein, APP Accumulation, and Enhanced Neuronal Plasticity

**Figure 4:**
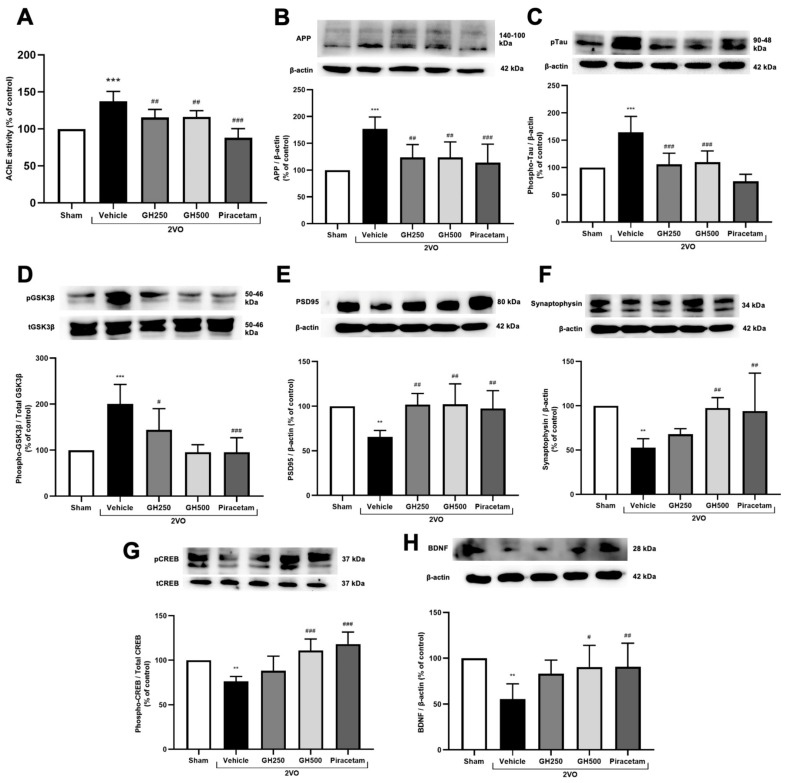
Gelatin hydrolysate attenuates AD markers in 2VO rats. (**A**) AChE activity. Western blotting was conducted to determine the protein expression of APP, pTau, phospho-GSK3β, Total-GSK3β, PSD95, synaptophysin, pCREB, Total CREB, and BDNF in 2VO rats. (**B**) Representative Western blot analysis band and quantification of the expression of APP, normalized to β-actin. (**C**) Representative Western blot analysis band and quantification of the relative expression of pTau, normalized to β-actin. (**D**) Representative Western blot analysis band and quantification of the expression of phospho-GSK3β, normalized to Total GSK3β. (**E**) Representative Western blot analysis band and quantification of the expression of PSD95, normalized to β-actin. (**F**) Representative Western blot analysis band and quantification of the expression of synaptophysin, normalized to β-actin. (**G**) Representative Western blot analysis band and quantification of the expression of pCREB, normalized to Total CREB. (**H**) Representative Western blot analysis band and quantification of the expression of BDNF, normalized to β-actin. All data are presented as mean ± SD. (*n* = 6 per group). ** *p* < 0.01 and *** *p* < 0.001 indicate a significant difference from the sham-operated group, and ^#^ *p* < 0.05, ^##^ *p* < 0.01, and ^###^ *p* < 0.001 denote a significant difference relative to the 2VO + vehicle group.

The pathologies of CCH, including learning and memory impairment, were assessed by measuring AChE activity, amyloid precursor protein (APP) expression, and tau protein phosphorylation. A significantly increased level of AChE activity was observed in 2VO rats compared to the sham group. GH or piracetam-treated rats showed significantly reduced AChE activity in comparison to 2VO (Figure 4A). Furthermore, the expression of APP was significantly elevated in 2VO rats compared to sham rats (*p* < 0.001). GH and piracetam treatments significantly decreased APP levels compared to the 2VO group (*p* < 0.01 for GH and *p* < 0.001 for piracetam). Tau protein dysfunction was evaluated by measuring the expression of phosphorylated tau. In 2VO rats, tau phosphorylation was significantly increased compared to the sham group (*p* < 0.001). However, the expression of phosphorylated tau was significantly decreased in 2VO rats treated with GH and piracetam (*p* < 0.001). Additionally, the expression of glycogen synthase kinase 3 β (GSK-3β), a key regulator of tau phosphorylation, was significantly upregulated in 2VO rats compared to sham rats (*p* < 0.001). Treatment with GH and piracetam significantly reduced GSK-3β levels (*p* < 0.05 for 250 mg/kg GH, *p* < 0.001 for 500 mg/kg GH and piracetam) (Figure 4B–D).

The expression of PSD95 and synaptophysin, key synaptic proteins in the cholinergic system, was assessed by Western blotting to evaluate cholinergic dysfunction associated with CCH study. In 2VO rats, the levels of PSD95 and synaptophysin were significantly decreased compared to the sham group (*p* < 0.01). However, treatment with GH and piracetam significantly increased the expression of both PSD95 and synaptophysin compared to the 2VO group (*p* < 0.01) (Figure 4E,F). Phosphorylated CREB, a major regulator of neurotransmitter and brain-derived neurotrophic factor (BDNF) levels, was also significantly lower in the 2VO group compared to sham (*p* < 0.01). Treatment with 500 mg/kg GH and piracetam resulted in significant increases in CREB phosphorylation (*p* < 0.001), which was associated with a significant increase in BDNF protein levels (*p* < 0.05 for 500 mg/kg GH and *p* < 0.01 for piracetam), compared to the 2VO group (Figure 4G,H). These results suggest that GH alleviates tauopathy and synaptic dysfunction in 2VO-operated rats.

## 3. Discussion

This study demonstrated the neuroprotective effects of GH against cognitive impairment and VaD induced by CCH. The key findings of this study revealed that GH enhanced antioxidant defenses, thereby mitigating CCH-induced oxidative stress and protecting against neuronal cell death. Furthermore, GH inhibited AD pathology by reducing the expression of APP, pTau, and pGSK3β, while promoting the activity of cholinergic neurons. GH also significantly improved synaptic function, as evidenced by the restoration of synaptic proteins, which correlated with observed cognitive improvements in behavioral tests.

This study used bigeye snapper skin GH, prepared by papain hydrolysis of bigeye skin gelatin (3% papain at 55 °C for 3 h), following the method described by Wangtueai et al. [21]. The GH yield and degree of hydrolysis (DH) were 69.36 ± 0.49% and 57.85 ± 0.82%, respectively. The crude GH exhibited strong DPP-IV inhibitory activity (IC50: 2.45 ± 0.02 mg/mL) and antioxidant activity (IC_50_ values for DPPH, ABTS, OH, and H_2_O_2_ radical scavenging were 2.38 ± 0.01, 1.99 ± 0.03, 0.25 ± 0.01, and 17.64 ± 0.37 mg/mL, respectively). This GH had higher antioxidant and DPP-IV inhibitory activities, which may be due to a higher content of low-MW bioactive peptides associated with a higher DH (57.85 ± 0.82%). This may be consistent with Li-Chan et al. [22], who reported that Atlantic salmon skin gelatin hydrolyzed with 6% flavorzyme had a DH of 42.5% and 45.2% DPP-IV inhibition at 5 mg/mL. The hydrolysate was divided into <1 kDa, 1–2.5 kDa, and >2.5 kDa fractions, with DPP-IV inhibition at 61.2%, 29.6%, and 43.2% (at 2 mg/mL), respectively. The <1 kDa were identified in Gly-Pro-Ala-Glu and Gly-Pro-Gly-Ala, with MWs of 372.4 and 300.4, respectively. Wang et al. [23] also found that low-MW (<1.5 kDa) fractions from halibut skin hydrolysate and tilapia skin gelatin hydrolysate showed DPP-IV inhibition of 38.2% and 51.9% at 1 mg/mL. In addition, previous studies reported that bigeye snapper gelatin contained high amounts of the main amino acids Gly and Pro, as well as a unique amino acid, Hyp [24,25]. Wang et al. [26] prepared salmon skin gelatin and hydrolysate via papain hydrolysis, yielding gelatin and hydrolysate with a typical amino acid composition rich in Gly, Pro, and Hyp. In this study, the crude bigeye snapper skin gelatin hydrolysate (GH) is used, and we provide basic information on GH characteristics as mentioned above. However, the molecular weight distribution, amino acid composition, and peptide sequence will be included in future work.

Cerebral hypoperfusion is a critical factor in the pathogenesis of VaD [2]. The resulting reduction in blood flow leads to an insufficient oxygen and nutrient supply to neuronal cells, creating a hypoxic environment [27,28]. Previous studies have demonstrated that blood supply to the cerebral cortex is reduced by 35–45% following 2VO [29]. HIF-1α, a transcription factor stabilized under low-oxygen conditions, translocates to the nucleus upon stabilization, where it promotes the expression of various target genes, including GLUT3 [30]. Increased GLUT3 expression facilitates enhanced glucose uptake to meet metabolic demands under hypoxic conditions. Consistent with previous studies, our results show that both HIF-1α and GLUT3 were upregulated in the 2VO group under CCH conditions. Notably, our data demonstrated that administration of GH and piracetam effectively modulated hypoxic conditions by reducing the expression of HIF-1α and GLUT3.

A primary consequence of hypoxia is the overproduction of ROS, which contributes to oxidative stress. It has been well-documented that oxidative stress plays a pivotal role in the progression of neurodegenerative disorders, including VaD [2]. In the brain, NADPH oxidases are a major source of ROS production, with NOX4 being particularly prominent in CCH due to its continuous generation of ROS [31,32,33]. In our study, we observed the levels of ROS, as well as the expression of 4-HNE and NOX4. Our results show that GH and piracetam effectively suppressed ROS levels and the expression of both 4-HNE and NOX4 proteins. In response to oxidative stress, the brain activates antioxidant defense mechanisms, one of which involves the Nrf2. Nrf2 is a transcription factor that regulates the expression of antioxidant enzymes, including SOD [5]. Previous studies have shown that CCH impairs Nrf2 expression, leading to an imbalance in oxidative stress and redox homeostasis [34]. A high level of oxidative stress under prolonged CCH conditions induces neuronal apoptosis, particularly in hippocampal CA1 neurons, which are highly sensitive to oxidative damage, thereby contributing to cognitive impairment [35,36]. Consistent with this, our results demonstrated that SOD was impaired in the vehicle group. However, treatment with GH and piracetam significantly increased SOD activity, possibly via activation of the Nrf2 pathway. In summary, our findings provide the first evidence that GH effectively modulates oxidative stress-related markers by enhancing antioxidant defenses, suggesting a potential therapeutic approach for managing CCH-induced cognitive impairment in VaD.

Collective evidence indicates that uncontrolled oxidative damage ultimately contributes to neuronal cell death [8,37]. To further elucidate the mechanisms underlying the neuroprotective effects of GH, we evaluated apoptotic markers. In the vehicle group, neuronal injury was observed, as evidenced by the presence of pyknotic nuclei in H&E-stained brain sections. In contrast, treatment with GH and piracetam markedly attenuated neuronal damage. At the molecular level, we investigated key apoptotic markers, pro-apoptotic proteins Bax and cleaved caspase-3. Our findings revealed that expression of pro-apoptotic markers was significantly elevated in the 2VO group, while GH and piracetam treatment suppressed their expression. Our results suggest that GH effectively protects against neuronal injury by inhibiting the apoptotic cell death pathway.

The hypoxic and oxidative environment associated with CCH is closely linked to the upregulation of AD markers, including amyloid plaque formation, tau hyperphosphorylation, and cholinergic dysfunction [2,27,28]. Although AD and VaD share overlapping features of cognitive impairment, the mechanistic distinctions between the two remain incompletely understood. Several studies have proposed that CCH impairs clearance in the brain by reducing cerebral blood flow, thereby hindering the removal of toxic metabolites such as amyloid-beta [38]. Moreover, HIF-1α has been identified as a mediator of AD pathology through its ability to induce BACE1, the enzyme responsible for Aβ generation [39]. Accumulating evidence supports the notion that CCH contributes to AD pathology by promoting Aβ and pTau accumulation, as well as increased AChE activity [27,40]. In this study, we demonstrate for the first time that GH modulates key hallmarks of AD pathology, including AChE activity, pTau, and APP. At the mechanistic level, we further examined the pGSK3β/GSK3β ratio, a critical regulator of tau phosphorylation [41]. Our results showed that GH treatment significantly reduced the pGSK3β/GSK-3β ratio, thereby attenuating tau hyperphosphorylation in the CCH-induced VaD model.

Accumulative data have shown that oxidative stress and apoptosis lead to the loss of synaptic connections [28,42,43]. GH effectively modulated pre- and postsynaptic markers, synaptophysin, and PSD95. Previous studies have shown that pCREB is impaired, affecting synaptic growth and survival [31,40]. Similarly, BDNF expression and its signaling pathways are disrupted. Therefore, we then measured the proteins related to neuronal plasticity, such as pCREB and BDNF. The results showed that the proteins involved in neuronal plasticity were decreased in the 2VO group, whereas high doses of GH improved neuronal plasticity, as denoted by promoting the expression of pCREB and BDNF. Therefore, our findings demonstrate that GH effectively protects synaptic transmission and signaling to promote synaptic plasticity.

The pathological changes following CCH-induced VaD exacerbate cognitive deficits. Our results are consistent with previous studies, as evidenced by the impairment in learning function due to a longer escape latency and in memory function due to a decreased time in the target quadrant and platform crossing [27,44]. Likewise, in the most recent studies, treatment with piracetam enhanced cognitive deficits as shown by the reversal of these parameters to the sham level [27,31,40]. Interestingly, our findings revealed for the first time that GH is capable of improving learning and memory deficits compared to the vehicle group. Therefore, our results suggest that GH and piracetam could attenuate the cognitive impairments in CCH-induced VaD. Recent studies indicate that oxidative stress activates inflammation-related signaling pathways, thereby further promoting neuronal injury and cognitive dysfunction [2]. Therefore, it is of interest to investigate the effects of GH on this mechanism in future studies.

## 4. Materials and Methods

### 4.1. Preparation of Fish Skin GH

Fish skin GH was prepared from the skin of bigeye snapper (*Priacanthus tayenus*) using the method of Wangtueai et al. [21]. Fresh fish skins were obtained from the fish fillets and fish mince processing plant at Samut Sakhon Province, Thailand. To prepare the fish skins, they were washed under running tap water to remove foreign matter, then packed in a zip-lock plastic bag (1000 g/bag) and stored at −18 °C to −20 °C until further use (not over 2 months). For fish gelatin extraction, frozen fish skins were thawed, washed in running tap water, and cut into small pieces by hand. The skins were then soaked in 5 vol (*v*/*w*) of 0.2 mol/L NaOH solution at 4 °C for 3 times with fresh solution each time. After soaking, they were drained, washed with running tap water until the pH was about 7, and soaked in 5 vol (*v*/*w*) of 0.05 mol/L CH_3_COOH solution for 3 h. The pretreated fish skins were rewashed under running tap water until the pH became ~7. Gelatin extraction was done in 2 vol (*v*/*w*) of distilled water at 50 °C for 12 h. The extracted solution was filtered through two layers of cheesecloth and filter paper (No. 1, Whatman, Maidstone, UK). The resulting filtrate was freeze-dried (GFD-3H freeze-dryer, Gririanthong, Samut Sakhon, Thailand) to obtain fish skin gelatin. To prepare the fish skin GH, fish skin gelatin was dissolved in distilled water at 5% (*w*/*v*), heated up until reaching the optimum temperature of the enzyme (55 °C), added 3% (*w*/*w* protein) of papain (Merck KGaA, Darmstadt, Germany), and hydrolyzed for 3 h. The hydrolyzed mixture was boiled at 95 °C for 10 min to stop the enzymatic reaction, cooled, and freeze-dried to obtain a powder of fish skin GH.

### 4.2. The 2-Vessel Occlusion Model

The 2VO model was used to induce chronic hypoperfusion in rats by bilateral common carotid artery occlusion following a previous study [45,46]. Rats were anesthetized by 10 mg/kg xylazine (Thai Meiji Pharmaceutical, Bangkok, Thailand) and 30 mg/kg Zoletil (Virbac, Carros, France) via intraperitoneal injection. Both sides of the common carotid artery were indicated and double-bound by USP 2-0 non-absorbable surgical suture (UNIK, Taiwan). Sham rats were similarly operated on, except that the arteries were strapped. The wound was stitched with the synthetic absorbable suture coated with VICRYL (3–0). Finally, all operated rats were allowed to rehabilitate for 24 h.

### 4.3. Experimental Animals

Male Wistar rats (weighing 250–280 g) were obtained from Nomura Siam International, Bangkok, Thailand. The rats were housed under controlled environmental conditions (25  ±  1 °C, 12 h light/dark cycle) with free access to a standard pellet diet and tap water. The Animal Ethics Committee approved the study under the guidelines for the care and use of laboratory animals under the Faculty of Medicine, Chiang Mai University (Permit no 32/2556).

After a one-week acclimatization period, the rats were randomly divided into five groups, with 15 rats in each group: (1) sham, (2) 2-vessel occlusion (2VO) model, (3) 2VO + 250 mg/kg GH, (4) 2VO + 500 mg/kg GH, and (5) 2VO + 600 mg/kg piracetam (Nootropyl, UCB PHARMA S.A., Braine-I’Alleud, Belgium) as a positive control. GH and piracetam were diluted in 0.9% normal saline solution and administered via oral gavage for 35 days following the 2VO surgery. At the end of the experiment, brain tissues were collected and stored at −80 °C.

### 4.4. Morris Water Maze (MWM) Test

The MWM test, initiated on postoperative day 30, was used to evaluate long-term spatial learning and memory in rats [47,48]. Rats in each group underwent MWM testing for six consecutive days. The apparatus consisted of a black circular pool (210 cm in diameter) filled with water maintained at 25 ± 1 °C, with a water depth of 51 cm. The pool was divided into four quadrants, and a hidden platform was submerged 2 cm below the water surface. To assess spatial learning during the first five days, rats were released from different starting positions, randomly assigned to the North (N), East (E), Southeast (SE), and Northwest (NW) points, and were required to locate the hidden platform located in the Southwest (SW) quadrant. The latency to reach the platform was recorded, with a maximum trial duration of 2 min. On day 6 (probe trial), the platform was removed to assess long-term memory, and each rat was allowed to explore the pool for 2 min starting from the NE position. Latency, time spent in the target (platform) zone, and the number of platform crossings were recorded and analyzed using Smart Tracking Software (version number: 3.0, Panlab Harvard Apparatus, Bioscience Company, MA, USA).

### 4.5. Nissl Staining

The brains were carefully and rapidly removed and fixed in 4% paraformaldehyde. After dehydration with a series of ethanol, the brain tissues were embedded in paraffin. Paraffin-embedded brain tissues were sectioned in the coronal plane at a thickness of 4 µm. The sections were subjected to Nissl staining. The brain sections were randomly selected to count the number of neuronal cells in the cortex, dentate gyrus, and CA1 areas by the Nissl staining technique (6*n* per group). Stained sections were then examined and photographed under a light microscope (Olympus BX51, Tokyo, Japan). The mean count per field was used for statistical analysis.

### 4.6. Brain Tissue Preparation

Whole-brain tissues were homogenized in an ice-cold lysis buffer containing 1 mM phenylmethyl sulfonylfluoride (PMSF), then centrifuged at 15,000 rpm for 15 min at 4 °C. The resulting supernatant was collected and stored at −80 °C.

### 4.7. Acetylcholinesterase Assay

The activity of AChE was measured using the standard Ellman’s method [49,50]. Brain homogenate was incubated with 5,5′-dithiobis nitrobenzoic acid, followed by acetylthiocholine iodide and 0.01 M sodium phosphate buffer (pH 8.0) at 37 °C. Change in the absorbance was measured at 405 nm every 1 min for 20 min by using a microplate reader (Bio-TEK Synergy H4, Agilent, Winooski, VT, USA).

### 4.8. Determination of Reactive Oxygen Species (ROS)

DCFH-DA sensitive dye was used to evaluate the level of intracellular ROS [51]. The brain tissue lysate was mixed with H_2_DCF-DA solution at 37 °C for 25 min. The levels of DCF fluorescence were measured at the excitation wavelength of 485 nm and the emission wavelength of 538 nm using a microplate reader (Bio-TEK Synergy H4, Agilent, Winooski, VT, USA).

### 4.9. Determination of Nitric Oxide (NO) Level

The levels of NO were determined by the Griess assay [52]. The brain lysate was mixed with 1% sulfanilamide in 5% phosphoric acid and 0.1% N-1-naphthylethylenediamine dihydrochloride in an equal volume and incubated at room temperature for 10 min. The absorbance at 540 nm was detected using a microplate reader (Bio-TEK Synergy H4, Agilent, Winooski, VT, USA).

### 4.10. Determination of Superoxide Dismutase (SOD) Activity

The SOD activity in brain tissues was measured using the SOD detection kit (Sigma-Aldrich, Darmstadt, Germany) in line with the manufacturer’s instructions. A microplate reader (Bio-TEK Synergy H4, Agilent, CA, USA) was then utilized to measure the absorbance at 450 nm.

### 4.11. Western Blot Analysis

The protein quantification in brain tissue lysate was done by Bradford assay (Bio–Rad, Hercules, CA, USA). The equal amounts of protein (25 mg) of each group were separated in a 12% TGX FastCast acrylamide gel (Bio–Rad, Hercules, CA, USA) and transferred to a polyvinylidene difluoride (PVDF) membrane (Immobilon-P, Millipore, Billerica, MA, USA). Following a 2 h blocking period at room temperature with 5% (*w*/*v*) nonfat milk, the membranes were subjected to overnight incubation at 4 °C with primary antibodies: anti-NOX4, anti-Glut3, anti-Nrf2, anti-HIF 1, anti-4 HNE, anti-Bcl-2, anti-Bax, anti-APP, anti-phospho-Tau, anti-phospho-GSK3β, anti-GSK3β, anti-BDNF, anti-PSD95, anti-phospho-CREB, anti-CREB, anti-synaptophysin, and anti-cleaved caspase-3 at 4 °C overnight. The β-actin protein was used for internal control. The horseradish peroxidase-conjugated secondary antibodies were used to probe the membrane for 2 h at room temperature. The blots were visualized with an enhanced chemiluminescence system (Merck Millipore, Darmstadt, Germany). The Chemiluminescent bands were visualized using a Chemi-Doc AppleGen system and quantitatively analyzed utilizing Image J software (version 1.54g).

### 4.12. Statistical Analysis

Data are expressed as mean ± standard deviation (SD). To test differences between multiple groups, a one-way ANOVA with Dunnett’s post hoc test was used, while escape latency results were analyzed by a two-way ANOVA followed by a Bonferroni posttest. Statistical significance was established when the *p*-value was <0.05.

## 5. Conclusions

In the present study, we investigated the underlying mechanism of GH in attenuating the pathological changes after CCH-induced VaD. Our findings noted that GH exerts neuroprotection and improves cognitive functions by modulating hypoxic response and antioxidant defense; by suppressing CCH-induced oxidative stress and neuronal cell death; by attenuating the AD markers; and by activating synaptic plasticity. However, some limitations of this study should be acknowledged. In particular, short-term memory was not independently evaluated in this study.

## Data Availability

The data supporting the findings of this study are available on request from the corresponding author.

## Abbreviations

The following abbreviations are used in this manuscript:

2VO	2-vessle occlusion
4-HNE	4-hydroxynonenal
AChE	Acetylcholinesterase
AD	Alzheimer’s disease
APP	Amyloid precursor protein
Aβ	Beta-amyloid
BDNF	Brain-derived neurotrophic factor
CCH	Chronic cerebral hypoperfusion
CREB	cAMP response element-binding protein
DCFH-DA	2′7′-Dichlorodihydrofluorescein diacetate
GH	Gelatin hydrolysate
GLUT3	Glucose transporter 3
GSK-3	Glycogen synthase kinase3
HIF-1	Hypoxia-inducible factor
MWM	Morris water maze
NO	Nitric oxide
NOX4	NADPH oxidase 4
Nrf2	Nuclear factor E2-related factor 2
PSD95	Postsynaptic density protein 95
pTau	Tau protein
ROS	Reactive oxygen species
SOD	Superoxide dismutase
VaD	Vascular dementia
